# Effect of Different Green Extraction Methods and Solvents on Bioactive Components of Chamomile (*Matricaria chamomilla* L.) Flowers

**DOI:** 10.3390/molecules25040810

**Published:** 2020-02-13

**Authors:** Jana Šic Žlabur, Ivanka Žutić, Sanja Radman, Maja Pleša, Mladen Brnčić, Francisco J. Barba, Gabriele Rocchetti, Luigi Lucini, Jose M. Lorenzo, Rubén Domínguez, Suzana Rimac Brnčić, Ante Galić, Sandra Voća

**Affiliations:** 1 Faculty of Agriculture, University of Zagreb, Svetošimunska Cesta 25, 10000 Zagreb, Croatia; jszlabur@agr.hr (J.Š.Ž.); izutic@agr.hr (I.Ž.); sradman@agr.hr (S.R.); maja00plesa@gmail.com (M.P.); agalic@agr.hr (A.G.); svoca@agr.hr (S.V.); 2 Faculty of Food Technology and Biotechnology, University of Zagreb, Pierottijeva ulica 6, 10000 Zagreb, Croatia; mbrncic@pbf.hr (M.B.); srimacbrncic@pbf.hr (S.R.B.); 3 Nutrition and Food Science Area, Preventive Medicine and Public Health, Food Science, Toxicology and Forensic Medicine Department, Faculty of Pharmacy, Universitat de València, Avda. Vicent Andrés Estellés, s/n 46100 Burjassot, València, Spain; francisco.barba@uv.es; 4 Department for Sustainable Food Process, Università Cattolica del Sacro Cuore, Via Emilia Parmense 84, 29122 Piacenza, Italy; gabriele.rocchetti@unicatt.it (G.R.); luigi.lucini@unicatt.it (L.L.); 5 Centro Tecnológico de la Carne de Galicia, Adva. Galicia n° 4, Parque Tecnológico de Galicia, 32900 San Cibrao das Viñas, Ourense, Spain; rubendominguez@ceteca.net

**Keywords:** German chamomile, ultrasound, conventional extraction, bioactive compounds, antioxidant capacity

## Abstract

Chamomile (*Matricaria chamomilla* L.) dried flowers contain a group of interesting biologically active compounds such as sesquiterpenes, flavonoids, coumarins, vitamins, phenolic acids and glucosides. Therefore, the aim of the present study was to characterize the composition in bioactive compounds (specialized metabolites) present in water and ethanol extracts of chamomile flowers, together with monitoring the impact of different extraction techniques (conventional vs. ultrasound-assisted extraction (UAE)) on the parameters under investigation. UAE treatment significantly decreased the extraction time of bioactive compounds from herbal material. Polyphenolic compounds content and antioxidant capacity were significantly higher in UAE extracts. Moreover, solvent type had a significant impact on the specialized metabolites content, while the highest vitamin C and polyphenols content were recorded in 50% ethanol (*v*/*v*) extracts. Optimization of basic extraction factors: solvent type, temperature and technique is crucial for obtaining the extracts with the highest content of specialized metabolites and antioxidant capacity.

## 1. Introduction

Chamomile (*Matricaria chamomilla,* synonym *Matricaria recutita* (L.) Rauschert) is a popular aromatic, medicinal herb mostly used in therapeutic purposes. Dried flowers and essential oil are the most widely used products mainly because it has multiple medicinal properties from antiinflammatory, analgesic, antimicrobial and antispasmic to sedative [1]. The most common variety in use is German chamomile (*Matricaria chamomilla*), while from the Asteraceae family well known variety is also Roman chamomile (*Chamaemelum nobile*).

Mentioned varieties strongly differs both in morphological and chemical composition primarily in the content of biologically active compounds including essential oils and several polyphenols [2,3]. Aqueous and alcoholic (methanol and ethanol) extracts of chamomile are prepared from dried flowers and used as foods, like herbal teas or like tinctures mostly in pharmaceutical and medicinal purposes. Even, currently the most popular chamomile product in use is a form of herbal tea consumed over million cups per day [3,4]. Moreover, more recent studies highlight a significant anticancer properties of chamomile extracts including anti-proliferative and apoptotic activity in various human cancer cells with minimal effect on normal cells [5,6,7].

In the production of extracts from natural sources various isolation techniques are combined while more and more attention is gained to the techniques focused to the principles of “green chemistry”, which among other things assumes using environmentally safe and nontoxic solvents [8,9,10,11]. For the extraction of various phytochemicals, specifically polyphenolic compounds the efficient solvents are usually ethanol, methanol, glycerol and their water solutions [12,13,14,15]. However, more attention has been gained to the use of solvents Generally Recognized As Safe (GRAS), such as water [16].

As above mentioned according to the principles of “green chemistry”, innovative extraction techniques are more developed and general increasingly used for isolation of various phytonutrients [17]. For instance, ultrasound-assisted extraction (UAE) shows a number of advantages in the extraction of different chemical compounds from plant materials, from the increased yield of the compound to significant energy savings and time reduction [14,18,19].

Therefore, the aim of the present study was to characterize the composition in bioactive compounds present in water and ethanol extracts of chamomile flowers, together with monitoring the impact of different extraction techniques (conventional vs UAE) on the parameters under investigation.

## 2. Results and Discussion

### 2.1. Organic Acids and Physicochemical Properties of Chamomile Extracts

**Figure 1:**
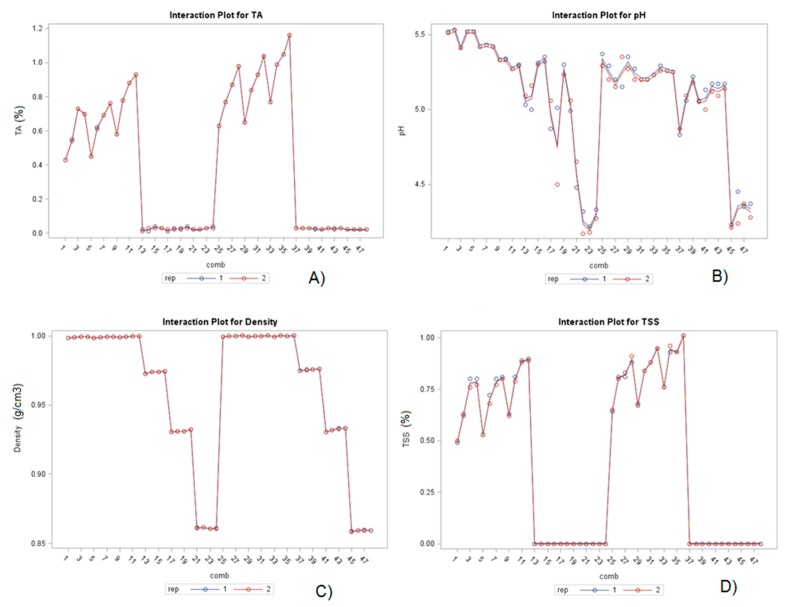
Interaction plots of physicochemical properties (**A**) total acids; (**B**) pH; (**C**) density and (**D**) total soluble solids of chamomile (cv. ‘Pitoma’) water (1–12 and 25–36) and ethanol extracts (13–24 and 37.48) in conventional (C1–C24) and UAE (U25–48) treatments.

Aromatic herbs are not characterized by a high content of organic acids, so low levels of total acid (TA) content determined in chamomile extracts regardless of the used solvent, extraction method or time is expected (Figure 1A). In aqueous extracts treated conventionally (C1–C12) the TA content was 0.67% while in ethanolic extracts (C13–C24) TA content was significantly lower (0.03%). In aqueous extracts, it was observed a significant positive impact of temperature and time for TA content since a higher temperature of extraction (60 °C) and longer extraction time (35 min) promoted an increased TA content. The highest TA content (0.93%) in aqueous extracts treated conventionally was determined in C12 treatment. On the other hand, in ethanolic extracts, alcohol concentration and time did not significantly influence TA content.

UAE treatment positively influenced (*p* < 0.001) on the TA content in aqueous extracts (U25–U36), in which was determined on average 32% higher TA content compared to the water extracts treated conventionally (Figure 1A). Moreover, longer extraction periods and higher temperature positively influenced on the TA content like in conventional extraction with the highest TA content (1.16%; sample U36).

Regarding ethanolic extracts, UAE did not significantly influence TA content since the average TA content for treatments U37–U48 was the same as for samples C13-C24. According to the determined low TA values for both water and ethanol extracts, relatively high pH values were observed in conventional and UAE extractions. The average pH value determined for aqueous extracts treated conventionally was 5.41 (C1–C12), for ethanol extracts (C13–C24) 4.84, while for aqueous extracts treated by UAE (U25–U36) 5.24 and for ethanolic extracts (U37–U48) 4.82.

As expected, density values significantly differed according to the temperature and ethanol concentration used. For aqueous extracts, the highest density both in conventional (0.9999 g cm^−3^) and UAE (1.0003 g cm^−3^) was determined in the sample treated using a temperature of 60 °C. Samples with higher ethanol (80%, U45–U48) had the lowest density (on average 0.8592 g cm^−3^) with no significant impact of the extraction method.

Likewise, total soluble solids (TSS) were only determined in aqueous extracts. Temperature increase (60 °C) positively influenced on the TSS content in the aqueous extracts, observing the highest values (0.90% and 1.01%, respectively) in the samples C12 and U36, respectively. Regardless of the water temperature and time period, UAE significantly influenced on the increase of the TSS content (about 16%) compared to the conventional extraction.

Different research studies suggested opposite results regarding the impact of UAE on the physicochemical parameters of liquid samples. For example, Aadil et al. [20] and Zou et al. [21,22] did not determine any changes of TA, TSS and pH values in UAE-treated samples, while other authors obtained a positive impact of UAE on the mentioned parameters [23]. The main reason for such significant variability in results found in the available literature is strongly correlated with the main condition parameters affecting the cavitation phenomenon during UAE. Namely, lower applied ultrasound frequencies results in the formation of bubbles with critical diameter, which easily collapse during compression cycles inducing the release of large amounts of heat and shockwaves, creating localized temperatures increase around 5000 K and pressure jets over 100 MPa, called transient cavitation [24,25]. Mild temperature increase in UAE systems as a direct effect of transient cavitation may affect some physical properties of the liquid such as density, TA, TSS and pH.

Interaction analysis of the main varied factors: extraction method (EM), solvent type (S) and time (t) in chamomile extracts is shown in Table 1. According to the obtained results, the interaction between factors EM × S significantly influenced on the all observed physicochemical parameters, while interaction between S × t did not influence on the pH and density. Interaction between EM × t did not significantly influence to any of the analyzed parameters. Moreover, the interaction between all the varied factors in the present study research (EM × S × t) significantly affected all the analyzed parameters.

### 2.2. Bioactive Content and Antioxidant Capacity of the Different Chamomile Extracts

**Figure 2:**
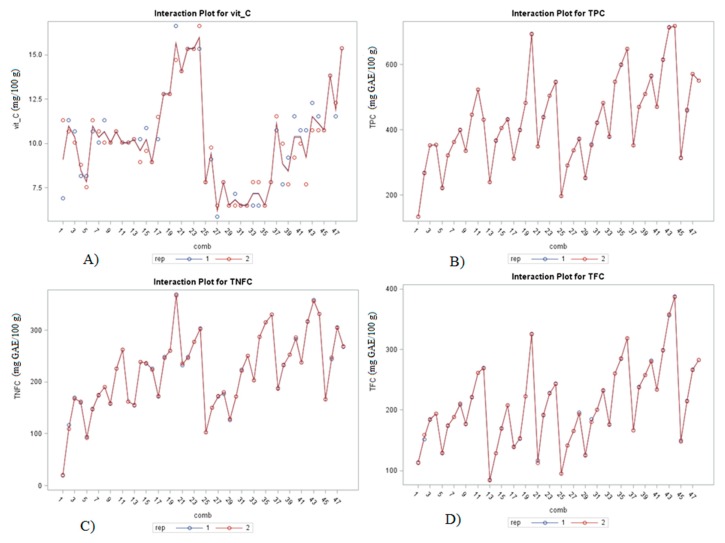
Interaction plot of vitamin C (**A**), total phenol (TPC; **B**), total non-flavonoid content (TNFC; **C**) and total flavonoid (TFC; **D**) content in chamomile (cv. ‘Pitoma’) water (1–12 and 25–36) and ethanol extracts (13–24 and 37.48) in conventional (C1–C24) and UAE (U25–48) treatments.

Regardless of the solvent type and time, significantly lower vitamin C content was determined in the UAE-treated samples (U25–U48; Figure 2A). On average, after conventional extraction, the vitamin C content of aqueous extracts was 25% higher compared to the UAE-treated extracts. Moreover, higher concentrations of ethanol (80%) significantly contributed to an increase of vitamin C content in chamomile extracts compared to the other solvents (H_2_O 21.4, 40 and 60 °C and EtOH of 20% and 50% *v*/*v*). The highest vitamin C content (15.98 mg, 100 g^−1^) was determined for the sample C24, while the lowest values (6.18 mg, 100 g^−1^) were found for the sample U27. The phenomenon of transient cavitation initiates chemical reactivity through thermolysis, supercritical water oxidation and free radical oxidation [24]. Impact of thermolysis but also the formation of hydrogen ions (H^+^), free radicals (O^−^, OH^−^ and HO_2_^−^) and hydrogen peroxide (H_2_O_2_) might be a possible explanation for the slight vitamin C degradation noticed in chamomile extracts in the present research. Most of literature data cited a positive effect of UAE on the vitamin C content but also some of them emphasized about the possibility of vitamin C degradation during sonolysis [26,27].

Besides the mentioned changes based on the chemical reactions occurring as a direct result of cavitation phenomena, sonication treatment (transient cavitation) also can promote a cell wall disruption, thus facilitating the release of various phenolic compounds. Chamomile extracts treated by UAE had significantly higher total phenol (TPC) content, even 20%, compared to the extracts conventionally treated (Figure 2B) regardless of the solvent type and time.

Both, after conventional and UAE extractions, TPC significantly differed between aqueous extracts prepared at different solvent temperatures with significantly higher TPC values recorded in extracts treated with higher temperature, 60 °C (C9–C12 and U33-U36) compared to the 21.4 (C1–C4 and U25–U28) and 40 °C (C5–C8 and U29–U32). In conventional treatments even 22% higher TPC and 8% in samples treated at 60 °C compared to the 21.4 and 40 °C, while in UAE even 74% and 35% higher TPC at 60 °C compared to the two other combined temperatures.

On the other hand, ethanol concentration significantly influenced TPC yield, independently of the extraction method used. In general, the highest TPC was determined in samples with 50% EtOH (*v*/*v*) compared to the samples with 20% or 80% EtOH. In UAE extracts with 50% EtOH (U41–U44), it was found a TPC even 33% higher compared to the extracts prepared with 20% and 80% EtOH. A similar trend was also observed for TPC after conventional extraction considering the EtOH concentration. Both in conventional (C20, 693.92 mg GAE 100 g^−1^) and UAE extraction (U44, 718.63 mg GAE 100 g^−1^) the highest TPC content was obtained in the extracts with 50% EtOH, and subsequent extraction for 35 min. The same trend observed for TPC was also found for total non-flavonoid (TNFC) and total flavonoid (TFC) compounds when the conventional and UAE extractions were compared.

In UAE chamomile extracts (U25-U48) was found with 18% higher TNFC (Figure 2C) and 23% TFC (Figure 2D) regardless of the solvent type and time compared to the extracts conventionally treated (C1–C24). A positive impact of higher solvent temperature (60 °C) in water extracts was also observed for the content of TNFC and TFC, both in conventional and UAE treatments. TNFC in water extracts conventionally treated with 60 °C H_2_O (C9–C12) was 33% higher compared to the extracts conventionally treated with 40 °C H_2_O (C5–C8) and even 74% higher compared to the extracts conventionally treated with H_2_O at room temperature (C1–C4).

In UAE extraction even higher positive temperature impact for TNFC was determined since even 47% higher values were determined in water extracts prepared with 60 °C H_2_O (U33–U36) compared to those prepared with 40 °C H_2_O (U29–U32) and 88% higher compared with extracts prepared with H_2_O at room temperature (U25-U28). The ethanol concentration of 50% (*v*/*v*) significantly contributed to the extraction of TNFC, both in conventional and UAE treatments since in ethanolic extracts prepared with 50% EtOH was determined the highest TNFC (C17–C20 and U41–U44). Higher TFC values were determined in water extracts prepared with 60 °C H_2_O (C9–C12), about 33% higher compared to the extracts prepared with 40 °C H_2_O (C5–C8) and 44% higher compared to the extracts C1–C4 (H_2_O room temperature) in conventional treatment, while 41% higher in extracts U33–U36 (60 °C H_2_O) compared to the those prepared with 40 °C H_2_O (U29–U32) and 75% compared to extracts prepared with H_2_O room temperature (U25–U28). Moreover, both conventional and UAE treatments had the highest TFC in the samples treated with 50% EtOH (Figure 2D).

Different previous research studies confirmed the positive impact of sonication treatment on the content of polyphenolic compounds: total phenols, flavonoids, phenolic acids, flavones, flavonols, etc. [21,23,28,29,30,31]. Namely, besides cell wall destruction as a direct mechanical result of transient cavitation, different reactions at the chemical level can occur during sonication [32,33]. During sonolysis, as before mentioned, free radicals: O^−^, OH^−^ and HO_2_^−^ are formed. OH^−^ radicals improve the functionality of polyphenolic compounds, thus increasing the hydroxylation degree and consequently as the hydroxylation degree is higher, functionality of polyphenols could be improved [34].

**Figure 3:**
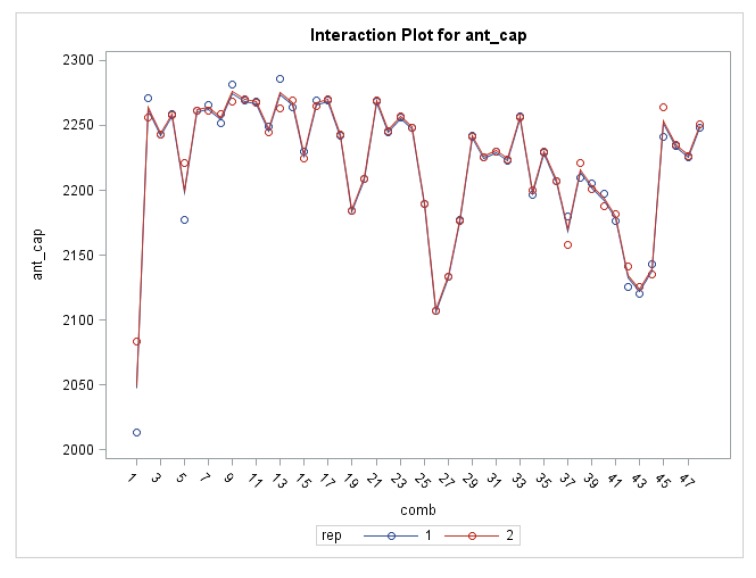
Interaction plot of antioxidant capacity (ant_cap) in chamomile (cv. ‘Pitoma’) water (1–12 and 25–36) and ethanol extracts (13–24 and 37.48) in conventional (C1–C24) and UAE (U25–48) treatments.

The analysis of the significance of the factor interactions in chamomile extracts for bioactive compounds and antioxidant activity (Figure 3) shows the highest significance (*p* ≤ 0.0001) in the interaction of all three varied variables: extraction method, solvent type and time, EM × S × t for all mentioned parameters (Table 2). Different combinations of factors, besides EM × S × t did not show any significance for specialized metabolites except for the extraction method and solvent type (EM × S) for TFC.

### 2.3. Multivariate Statistics to Discriminate the Different Extraction Conditions

The orthogonal projections to latent structures discriminant analysis (OPLS-DA) supervised approach was used in order to evaluate the impact of the different extraction conditions on the two chamomile cultivars (i.e., ‘Pitoma’ and ‘Bode Gold’). The OPLS-DA analysis allows us to identify which variables are driving the separation between two groups of observation. In particular, when considering the OPLS-DA score scatter plot, the horizontal direction of this plot will capture the variation between the groups, while the vertical dimension and any higher component of the so-called orthogonal type will capture variation within the groups. In our experimental conditions, the dataset based on pH, density, TSS, TA, alcohol volume content, vitamin C, TPC, TFC, TFNC and antioxidant capacity for both cultivars was used for building OPLS-DA models.

**Figure 4:**
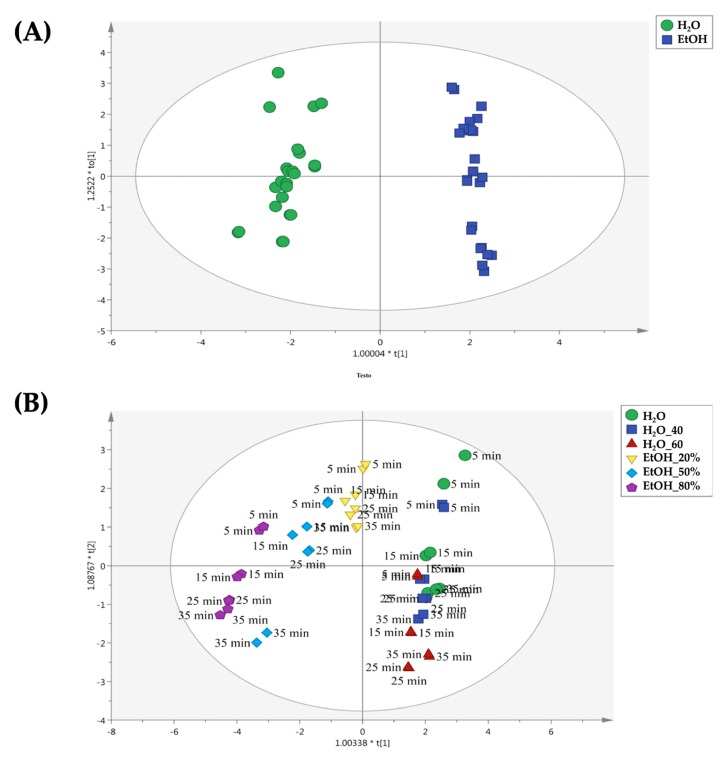
Orthogonal projections to latent structures discriminant analysis (OPLS-DA) to discriminate samples belonging to the Pitoma cultivar and extracted by the means conventional method. The score plots are built considering as class membership criteria both solvent type (**A**) and extraction time (**B**).

According to our experimental conditions, four OPLS-DA models were built. For both cultivars, the extraction solvent (i.e., hydroalcoholic vs water) was used as a class membership criterion, in order to identify the main differences during the four time periods considered (i.e., 5, 15, 25 and 35 min). The first OPLS-DA model is reported in Figure 4**,** regarding the use of conventional extraction method for the Pitoma cultivar. As can be observed from the Figure 4A, a clear separation between water and hydroalcoholic extracts was observed, thus confirming the different extraction efficiency in terms of solvent used. Afterwards, by inspecting each replicate of the OPLS-DA score plot (Figure 4B), it was clear that the extraction time was very effective in determining the differences in the profiles observed. In particular, we found that the most distinct profiles for conventional extractions were obtained when long extraction times were used (i.e., 25 and 35 min, respectively). Interestingly, the conventional extraction using ethanol 50% as solvent for 35 min, produced chemical profiles similar to those obtained by using ethanol 80% for 25 and 35 min.

**Figure 5:**
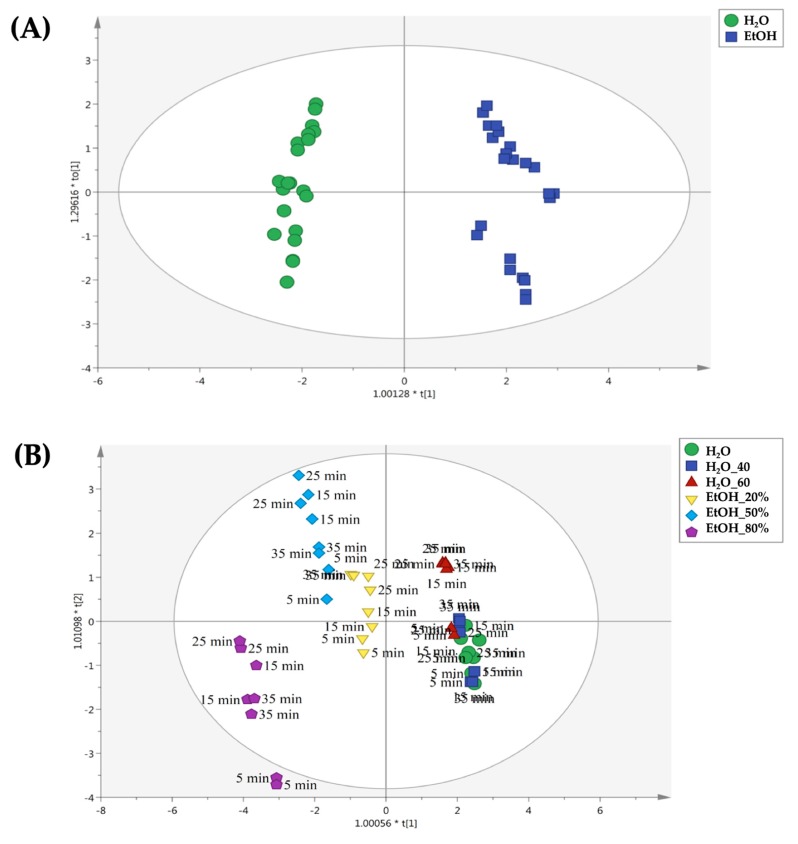
Orthogonal projections to latent structures discriminant analysis (OPLS-DA) to discriminate samples belonging to the Pitoma cultivar and extracted by means UAE. The score plots are built considering as class membership criteria both solvent type (**A**) and extraction time (**B**).

The second OPLS-DA model built considering the Pitoma cultivar samples extracted by UAE is reported in Figure 5. The same good separation between aqueous and alcoholic extracts was observed (Figure 5A). Interestingly, the OPLS-DA model on UAE treated samples allowed us to identify definitely different results when compared with conventional extracts. In fact, as shown in Figure 5B, we found that using ethanol 80% as extraction solvent for 5 min was able to produce the most differential profiles. Overall, the extraction time seemed to not play a crucial role in determining differences, when considering the water extracts. In fact, most of the differences for UAE water extracts were observed when using distilled water at 60 °C for 25 and 35 min. Therefore, the OPLS-DA models built for the Pitoma cultivar suggested that the combination of extraction solvent × extraction time should be carefully evaluated in order to promote differences in the chemical parameters investigated.

**Figure 6:**
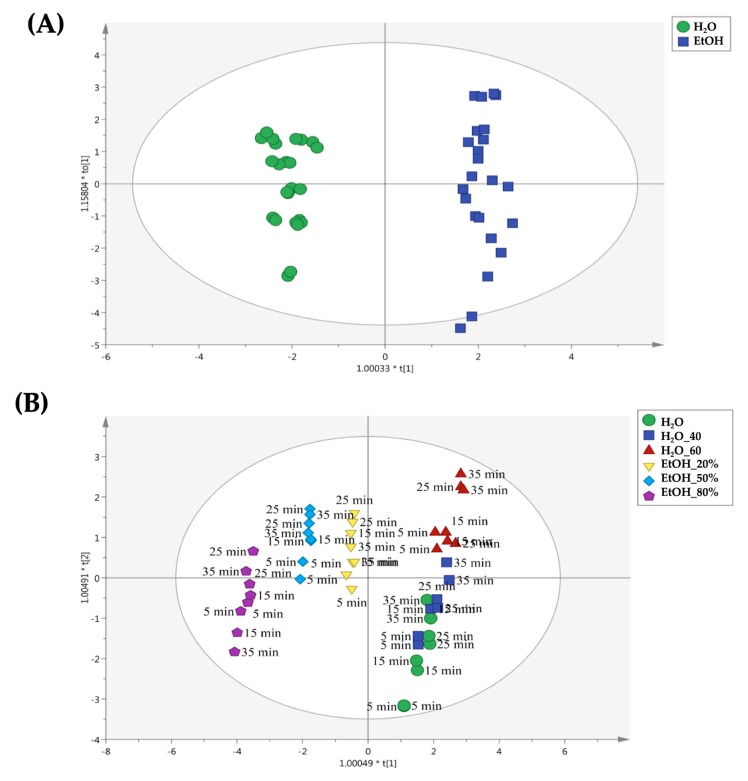
Orthogonal projections to latent structures discriminant analysis (OPLS-DA) to discriminate samples belonging to the Bode gold cultivar and extracted by the means conventional method. The score plots are built considering as class membership criteria both solvent type (**A**) and extraction time (**B**).

The third OPLS-DA model is reported in Figure 6, regarding the use of conventional extraction method for the Bode gold cultivar. As can be observed from the Figure 6A, similar results were obtained when comparing Pitoma and Bode gold score scatter plots. In this regard, a clear separation between water and hydroalcoholic extracts was obtained when considering the second latent vector of the predictive model, while the following OPLS-DA score plot (Figure 6B) built inspecting each replicate separately showed that ethanol 80% was the extraction solvent promoting the most different profile, independently from the extraction time considered. Overall, each different percentage (i.e., 20%, 50% and 80% of ethanol) allowed us to observe distinct signatures in the OPLS-DA score plot. Regarding water extracts, long extraction times combined with distilled water at 60 °C allowed us to observe the most discriminant chemical profiles.

**Figure 7:**
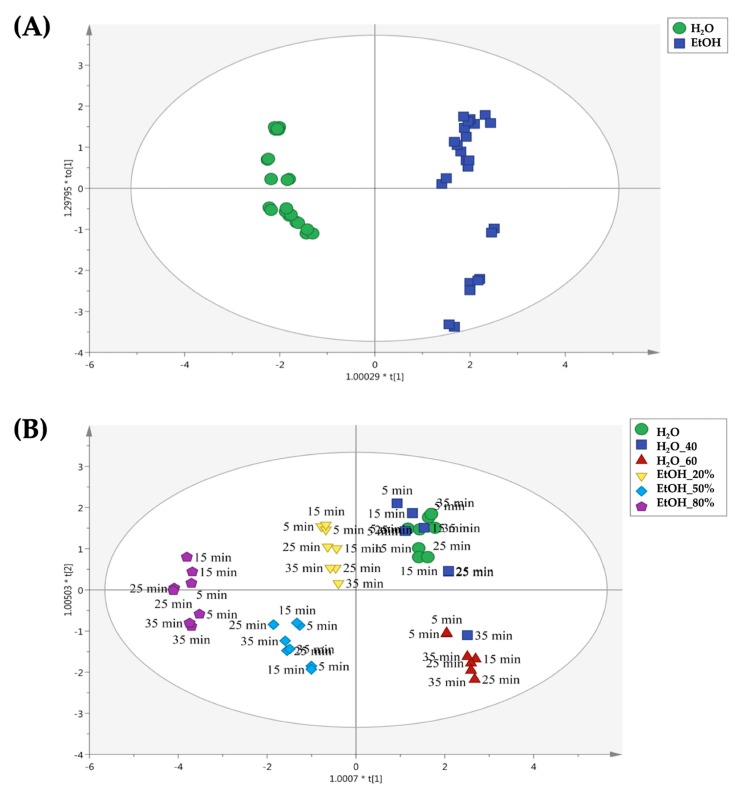
Orthogonal projections to latent structures discriminant analysis (OPLS-DA) to discriminate samples belonging to the Bode gold cultivar and extracted by means UAE. The score plots are built considering as class membership criteria both solvent type (**A**) and extraction time (**B**).

The fourth OPLS-DA model built considering Bode gold cultivar samples extracted by UAE is reported in Figure 7. It was found again a good separation degree between aqueous and hydroalcoholic extracts into the score scatter plot (Figure 7A). Interestingly, the OPLS-DA model on UAE treated samples showed definitely different trends when compared with conventional ones. In fact, as shown in Figure 7B, it was observed that using a different combination of ethanol as an extraction solvent (i.e., 20%, 50% and 80%) produced the most differential profiles, independently from the extraction time. Therefore, the selection of the extraction solvent seemed to be more important when compared to the extraction time applied. Overall, water extracts at room temperature were found to cluster with water extracts at 40 °C. Interestingly, samples extracted in water at 60 °C were grouped into the hyperspace together with those samples extracted with water at 40 °C for 35 min.

Finally, the variable selection method variables importance in projection (VIP) was used to identify those parameters allowing the score plot hyperspace distributions previously described (i.e., considering conventional and UAE extraction methods). The most discriminant parameters for both Pitoma and Bode gold cultivars are reported in Table 3; as can be observed, when considering the conventional extraction methods, the most discriminant and affected parameters were mainly: TSS, TA, volume content and density. However, using conventional extraction, vitamin C content was mainly affected in the Bode gold cultivar (VIP score = 1.07) when compared with Pitoma (VIP score < 1). Interestingly, the antioxidant capacity together with the parameters related to polyphenols (such as TPC and TFC) were not significantly affected by the two different extraction conditions (VIP scores < 1). The VIP markers found for UAE OPLS-DA models are reported also in Table 4 and considering both cultivars. The common variables most affected by the extraction conditions for UAE were TSS, TA, volume content and density. All the other parameters presented VIP scores lower than 1, except for vitamin C that was found to be very important in the OPLS-DA model built (VIP score = 1.08) considering the Pitoma cultivar.

## 3. Materials and Methods

### 3.1. Plant Material

For research purposes German chamomile (*Matricaria chamomilla*) cv. ‘Pitoma’ was cultivated. Sowing was conducted in the period from 15 October to 1 November 2016 in an area of 34 ha in Kutina, Croatia. Before sowing, basic agricultural practices were carried out: ploughing, soil milling and rolling. Since the cultivation is carried out according to the principles of organic production, mineral fertilizers and plant protection products were not used. Chamomile flowers were picked up by machine pulled combine on 26 May 2017 and dried. Drying was carried out at a temperature of 42–45 °C for 24 h. Dried flowers are packed in cardboard boxes and stored in a dry space until intended chemical analysis.

### 3.2. Preparation of Extracts

Preparation of chamomile extracts and all chemical analysis were conducted at the Department of Agricultural Technology, Storage and Transport at University of Zagreb Faculty of Agriculture. First, dried chamomile flowers were milled in laboratory mill (IKA MF-10, IKA®-Werke GmbH & Co., Staufen, Germany). The dry matter content (89.28%) of the chamomile flowers was determined using a conventional drying process at 105 °C [35]. For the extraction purposes (both conventional and ultrasonic-assisted) 100 mL of the following solvents: (i) distilled water at different temperatures (21.4, 40 and 60 °C) and (ii) ethanol (EtOH) at different concentrations (20%, 50% and 80%, *v*/*v*), were added into 2.5 g of dried milled chamomile flowers. Such prepared samples were extracted for 5, 15, 25 and 35 min after each filtered through Whatman filter paper. For the ultrasound-assisted extraction (UAE) immediately after the addition of different solvents, samples were placed in the ultrasonic bath (RK 103 H, Bandelin, Germany) frequency of 35 kHz, maximal nominal output power of the device 140 W and sonicated in the same time intervals (5, 15, 25 and 35 min). It should be noted that during UAE with distilled H_2_O at elevated temperatures (40 and 60 °C) the temperature in the ultrasonic bath was set up and maintained for 40 and 60 °C. During sonication, the temperature in samples was measured with a laser thermometer (Raytek–MiniTemp FS, Raytek, Toronto, ON, Canada) in time intervals of 30 s. The maximum recorded temperature during sonication of water extracts (was 64 °C for sample sonicated for 35 min and distilled H_2_O at 60 °C, while for the ethanol extracts the maximum recorded temperature during sonication was 47 °C for samples sonicated for 35 min and EtOH (80%, *v*/*v*) as the solvent. The experimental design set up for conventional and UAE is shown in Table 4.

### 3.3. Determination of Physicochemical Properties of Extracts

The physicochemical properties of the aqueous and ethanolic chamomile extracts included the determination of (i) density (g cm^−1^), (ii) total soluble solids content (TSS, %) only in water extracts, (iii) alcohol volume content (vol. %) only in ethanol extracts by digital densitometer (Densito 30PX, Mettler-Toledo, Switzerland); (iv) pH value using a digital pH-meter (Sevenmulti, Mettler Toledo, Switzerland) and (v) total acid content (TA, %), by potentiometric titration according to the AOAC [35].

### 3.4. Determination of Antioxidant Bioactive Components

The analysis included the determinations of the vitamin C content (mg 100 g^−1^) by titration with 2,6-dichlorindophenol according to AOAC [36]; total phenol content (TPC) according to the Shukla et al. [37] as follows: 1 mL of the extract and 1 mL of the Folin–Ciocalteu reagent diluted with distilled water (1:2) were added in a volumetric flask with a volume of 50 mL and allowed to stand for 3 min. Additionally, 3 mL of a saturated sodium carbonate solution was added, the flask was filled to the mark with distilled water and allowed to stand for 3 h at room temperature with intermittent shaking. The absorbance of the blue color was measured spectrophotometrically (Shimadzu UV 1650 PC, Shimadzu Co., Kioto, Japan) at 750 nm with distilled water as a blank; determination of total flavonoids content (TFC) according to the Abou-Arab and Abou-Salem [38] as follows: 1 mL of the extract, 1 mL of 20% HCl (*v*/*v*) and 0.5 mL formaldehyde were added in a volumetric flask volume of 25 mL. The prepared samples were blown with nitrogen (N_2_) and allowed to stand for 24 h at room temperature. After 24 h, the same Folin–Ciocalteu reaction as for the total phenols was carried out. As an external standard for TPC gallic acid, while for TFC catechin was used and the final content of TPC and TFC was expressed as mg GAE 100 g^−1^. Total non-flavonoid content (TNFC, mg GAE 100 g^−1^) was mathematically expressed as difference between TPC and TFC.

### 3.5. Determination of Antioxidant Capacity Using the ABTS Assay

The antioxidant capacity of the chamomile extracts was determined by the ABTS method according to the Miller et al. [39] and Re et al. [40]. As an antioxidant standard Trolox was used, while for the preparation of stock solution 2.5 mM Trolox was prepared in ethanol (80%). For the preparation of the stable ABTS radical solution (ABTS^•+^), 5 mL of ABTS solution (7 mM) and 88 µL of potassium persulfate (140 mM) solution were mixed and allowed to stand in the dark at room temperature for 16 h. On the day of analysis, 1% ABTS^•+^ solution (in 96% EtOH) was prepared. A 160 µL of extract was directly injected in the cuvette and mixed with 2 mL 1% ABTS^•+^. After 5 min, the absorbance at 734 nm was measured (Shimadzu 1650 PC, Germany). The final results of the antioxidant capacity was calculated based on calibration curve and expressed as mmol TE L^−1^. ABTS, 2, 2′-azinobis (3-ethylbenzothiazoline-6-sulfonic acid), Trolox (6-hydroxy-2,5,7,8-tetramethylchroman-2-carboxylic acid) and potassium persulfate were supplied from Sigma-Aldrich (St. Louis, MO, USA).

### 3.6. Statistical and Multivariate Analyses

All treatments, as for the conventional and UAE extraction, were made in triplicate. Chemical analysis was performed in duplicate. Generalized linear model, including repetition, extraction method (conventional, UAE), solvent type (H_2_O, EtOH) and time (5, 15, 25 and 35 min) as categorical predictors, was used. For the analysis procedures PROC GLM in SAS software package, version 9.3. was used. The obtained data were analyzed using an analysis of the variance (ANOVA). Mean values were compared by the *t*-test (LSD) and were considered significantly different at *p* ≤ 0.0001. In Tables different letters are shown to indicate significant differences between mean values within each column and also standard deviation (±SD) was expressed.

Afterwards, the whole dataset (considering both cultivars) was exported into SIMCA 13 (Umetrics, Malmö, Sweden) and elaborated by means of supervised orthogonal projections to latent structures discriminant analysis (OPLS-DA). In particular, the variation between groups was separated into predictive and orthogonal components. The OPLS-DA score plots allowed us to observe similarities between the different treatments, then describing the parameters better depicting both differences and similarities. To validate the OPLS-DA model, the R^2^Y and Q^2^Y were inspected, together with Hotelling’s T2 (using 95% and 99% confidence limits for suspect and strong outliers, respectively). Each OPLS-DA model built was cross-validated (CV-ANOVA; *p* < 0.01) and permutation testing (*n* = 100) was then carried out to exclude overfitting of the models. Finally, the most discriminant variables characterizing each model built were outlined by means of variables importance in projection (VIP) approach. The parameters possessing the highest discrimination potential were those possessing a VIP score > 1.

## 4. Conclusions

Comparing the impact of treatment method (conventional vs. UAE) on investigated physicochemical properties, both of water and ethanol based extracts, could be concluded that the ultrasonic treatment had a positive effect on the total acid content, while other properties such as the density, total soluble solids and pH-value did not significantly change given the applied extraction method. The ultrasonic treatment exhibited a significant positive effect on the analyzed total phenol, flavonoid and non-flavonoid content. The highest total phenol content (718 mg GAE 100 g^−1^) was recorded in the ethanol based extract (50%, *v*/*v*) treated by ultrasound for 35 min. The ultrasonic treatment did not positively affect the vitamin C content since a slight degradation of vitamin C in extracts was recorded with ultrasonic application. Besides, multivariate statistics applied to the parameters under investigation allowed us to clearly discern between water and ethanol-based extracts, highlighting also a clear role of the cultivars under investigations in determining the observed differences. In addition, the most affected parameters as resulted by conventional vs UAE extraction methods were provided. In this regard, TSS, TA and volume content were found to possess the highest VIP scores, thus confirming their importance in discriminating Pitoma and Bode gold cultivars. Therefore, based on the obtained results, it is possible to conclude that prepared infusions and tinctures were a rich source of specialized metabolites, with the ultrasonic treatment as an effective tool for obtaining the increased yield of extracted compounds in a shorter time period, thereby simultaneously obtaining the final product with an increased nutritional value.

## Figures and Tables

**Table 1:** The significance of the factor interactions in chamomile extracts for analyzed physicochemical properties.

Significance of the Factor Interactions				
Factor Interactions	TA Pr ≤ F	pH Pr ≤ F	Density Pr ≤ F	TSS Pr ≤ F
EM × S	0.0001	0.0001	0.0001	0.0225
S × t	0.0005	0.0788	0.8988	0.0001
EM × t	0.9987	0.9290	1.0000	0.9974
EM × S × t	0.0001	0.0001	0.0001	0.0001

EM × S—extraction method and type of solvent; S × t—solvent and time; EM × t—extraction method and time; EM × S × t—extraction method, solvent and time.

**Table 2:** The significance of the factor interactions in chamomile extracts for specialized metabolites and antioxidant activity.

Significance of the Factor Interactions					
Factor Interactions	VIT_C Pr ≤ F	TPC Pr ≤ F	TNFC Pr ≤ F	TFC Pr ≤ F	Ant_act Pr ≤ F
EM × S	0.0088	0.2264	0.1166	0.0082	0.0537
S × t	0.3480	0.7632	0.6586	0.7108	0.1775
EM × t	0.6927	0.9780	0.9891	0.8958	0.1118
EM × S × t	0.0001	0.0001	0.0001	0.0001	0.0001

EM × S—extraction method and type of solvent; S × t—solvent and time; EM × t—extraction method and time; EM × S × t—extraction method, solvent and time.

**Table 3:** VIP (variables importance in projection) selection method to identify those parameters discriminating chamomile extracts according to the different cultivars and extraction method.

	Conventional		UAE	
Variable OPLS-DA	Pitoma	Bode Gold	Pitoma	Bode Gold
TSS	1.27 ± 0.11	1.27 ± 0.07	1.26 ± 0.25	1.34 ± 0.05
TA	1.26 ± 0.10	1.26 ± 0.05	1.25 ± 0.26	1.32 ±0.05
Volume content	1.14 ± 0.09	1.14 ± 0.05	1.12 ± 0.22	1.17 ± 0.04
Density	1.10 ± 0.11	1.10 ± 0.06	1.08 ± 0.23	1.14 ± 0.06
pH	1.02 ± 0.11	1.00 ± 0.14	0.92 ± 0.22	0.88 ±0.21
TFNC	1.00 ± 0.25	0.70 ± 0.31	0.84 ± 0.95	0.74 ± 0.19
Vitamin C	0.93 ± 0.10	1.07 ± 0.21	1.08 ± 0.26	0.80 ± 0.38
TPC	0.84 ± 0.28	0.77 ± 0.06	0.85 ± 0.95	0.77 ± 0.14
TFC	0.71 ± 0.23	0.82 ± 0.08	0.85 ± 0.92	0.81 ± 0.31
Antioxidant capacity	0.34 ± 0.98	0.55 ± 0.32	0.43 ± 1.00	0.69 ± 0.28

**Table 4:** Experimental conditions for conventional (CONV) and ultrasound-assisted extraction (UAE).

Solvent	Temperature (°C)	Concentration (%, *v*/*v*)	Time (min)	CONV	UAE
H_2_O	21.4	-	5	C1	U25
H_2_O	21.4	-	15	C2	U26
H_2_O	21.4	-	25	C3	U27
H_2_O	21.4	-	35	C4	U28
H_2_O	40	-	5	C5	U29
H_2_O	40	-	15	C6	U30
H_2_O	40	-	25	C7	U31
H_2_O	40	-	35	C8	U32
H_2_O	60	-	5	C9	U33
H_2_O	60	-	15	C10	U34
H_2_O	60	-	25	C11	U35
H_2_O	60	-	35	C12	U36
EtOH	-	20	5	C13	U37
EtOH	-	20	15	C14	U38
EtOH	-	20	25	C15	U39
EtOH	-	20	35	C16	U40
EtOH	-	50	5	C17	U41
EtOH	-	50	15	C18	U42
EtOH	-	50	25	C19	U43
EtOH	-	50	35	C20	U44
EtOH	-	80	5	C21	U45
EtOH	-	80	15	C22	U46
EtOH	-	80	25	C23	U47
EtOH	-	80	35	C24	U48
